# Dissociative symptomatology in cancer patients

**DOI:** 10.3389/fpsyg.2015.00118

**Published:** 2015-02-24

**Authors:** Cristina Civilotti, Lorys Castelli, Luca Binaschi, Martina Cussino, Valentina Tesio, Giulia Di Fini, Fabio Veglia, Riccardo Torta

**Affiliations:** ^1^Department of Psychology, University of TurinTurin, Italy; ^2^Fondazione Edo ed Elvo Tempia per la Lotta contro i TumoriBiella, Italy; ^3^Clinical Psychology and Psycho-Oncology Unit, Department of Neuroscience, University of TurinTurin Italy

**Keywords:** cancer, dissociative symptoms, post traumatic stress disorder, psychoncology, peritraumatic dissociation

## Abstract

**Introduction**: The utilization of the post-traumatic stress disorder (PTSD) diagnostic spectrum is currently being debated to categorize psychological adjustment in cancer patients.

The aims of this study were to: (1) evaluate the presence of cancer-related traumatic dissociative symptomatology in a sample of cancer patients; (2) examine the correlation of cancer-related dissociation and sociodemographic and medical variables, anxiety, depression, and post-traumatic stress symptomatology; (3) investigate the predictors of cancer-related dissociation.

**Methods**: Ninety-two mixed cancer patients (mean age: 58.94, ds = 10.13) recruited from two hospitals in northern Italy were administered a questionnaire on sociodemographic and medical characteristics, the Karnofsky Scale to measure the level of patient activity and medical care requirements, the Hospital Anxiety and Depression Scale (HADS) to evaluate the presence of anxiety and depression, the Impact of Event Scale Revised (IES-R) to assess the severity of intrusion, avoidance, and hypervigilance, and the Peritraumatic Dissociative Experiences Questionnaire (PDEQ) to quantify the traumatic dissociative symptomatology.

**Results**: 31.5% of participants report a PDEQ score above the cutoff. The results indicated that dissociative symptomatology was positively correlated with HADS scores (HADS-Anxiety: *r* = 0.476, *p* < 0.001; HADS-Depression: *r* = 0.364, *p* < 0.001) and with IES-R scores (IES-R-Intrusion: *r* = 0.698, *p* < 0.001; IES-R-Avoidance: *r* = 0.619, *p* < 0.001; IES-R- Hypervigilance: *r* = 0.681, *p* < 0.001). A stepwise regression analysis was performed in order to find the predictors of cancer-related traumatic dissociative symptomatology. The results converged on a three predictor model revealing that IES-R-Intrusion, IES-R-Avoidance, and IES-R-Hyperarousal accounted for 53.9% of the explained variance.

**Conclusion:** These findings allow us to hypothesize a specific psychological reaction which may be ascribed to the traumatic spectrum within the context of cancer, emphasizing the close relationship between the origin of dissociative constituents which, according to the scientific literature, compose the traumatic experience. Our results have implications for understanding dissociative symptomatology in a cancer population and can help develop clinical programs of prevention and support for patients.

## INTRODUCTION

Of the many life-threatening illnesses, cancer can be one of the most stressful that a person might have to go through, in spite of the progress in diagnosis, treatments, and screening. Although the scientific literature (Bultz and Holland, 2006) and International Guidelines (National Comprehensive Cancer Network [NCCN], 2013) increasingly focus on the importance of recognizing and managing psychological distress in cancer patients, the burden of the latter can very often still be unacknowledged and underdiagnosed (Fallowfield et al., 2001; Söllner et al., 2001). Patients who may overtly exhibit symptoms linked to mental distress may not receive adequate treatment (Morasso and Tomamichel, 2005; Guglielmucci et al., 2014), leading them to keep their emotional problems to themselves.

From a clinical perspective, cancer has several traumatizing aspects: it affects the quality of life and survival (Holzner et al., 2013), as well as subjective well-being (Tessier et al., 2012; Hou and Lam, 2014). It generates a sense of vulnerability, loss of control, and hopelessness (Rodin et al., 2009; Sarenmalm et al., 2013), as well as causing emotional reactions characterized by a high level of arousal, avoidance in everyday life, and intrusive thoughts that interfere with normal functioning (Roper, 2000).

The link between cancer and post-traumatic stress disorder (PTSD) has been reported in various scientific studies, even though the investigation of life-threatening diseases as a potentially traumatic outcome still remains at an early stage: today, there is still neither agreement nor a distinctive diagnostic line in common. In the psycho-oncological literature, some studies are less favorable regarding the possibility of cancer leading to PTSD (Mehnert and Koch, 2007; Greimel et al., 2013; Phipps et al., 2014), while others are more favorable (Posluszny et al., 2011; Kangas et al., 2013). A recent meta-analysis (Abbey et al., 2014) shows how the cancer experience is traumatic enough to induce PTSD in a significant minority of cancer survivors.

The most critical views have reservations about the appropriateness of the diagnosis of PTSD in patients with cancer. The DSM V (American Psychiatric Association [APA], 2013), continuing the DSM IV-TR (American Psychiatric Association [APA], 2000), reports a classification system mainly indicated for physically healthy patients with mental disorders.

Cancer differs from other traumatic events in several aspects: (a) the imminence and degrees of life-threatening consequences, because cancer is not a singular trauma and PTSD may operate in a different way in patients diagnosed with cancer compared to individuals with PTSD who have experienced a single traumatic event such as an accident or assault (Greimel et al., 2013); (b), cancer patients commonly experience future-oriented and anticipatory worries such as fear of recurrence linked to realistic and potentially life-threatening stressors (Mehnert et al., 2009); (c) the internality of threat that may affect the perception and meaning of the cancer threat, primarily in terms of its perceived inescapability (Gurevich et al., 2002). In the light of the foregoing, it is not possible to know in advance the impact of the disease experience on each individual nor is it easy to determine which answers can be placed in a framework of normality and which in one of pathology. It is important to reflect on what sense it makes to talk about post-traumatic stress when faced with such events, where no patient is ever declared “definitely cured” (Trotti and Bellani, unpublished).

Gurevich et al. (2002) reported that among patients with newly diagnosed cancer at an early stages only 3–4% have a full PTSD, while 20% show subthreshold symptoms of PTSD; this is then present in 35% of patients after treatment and in 80% of cases of recurrence. In support of the hypothesis on the importance of assessing the presence of post-traumatic clinical symptoms, rather than the overall picture, Blanchard et al. (1996), Stein et al. (1997), and Marshall et al. (2001) propose some methods for the detection of subthreshold PTSD, taking into account the responses to stress as located on a continuum, rather than distinguishing cases in which it is present or not.

From this perspective, the word trauma regains its original meaning of “wound” (τρα vμα), used not in the sense of a singular event, but as a “psychobiological wound,” evolved from psychological, biological, and social factors, and with different degrees and symptom paths in developing psychological pain. The factors involved in the genesis of a PTSD symptomatology could involve limitations of the patient’s integrative capacity, expressed, for example, with dissociative reactions, affect dysregulation, and persistent avoidance of traumatic memories (Nijenhuis and van der Hart, 2011).

Our study examines the presence of processes and dissociative states in a sample of cancer patients. It seems that dissociation occupies an important position in the genesis of emotional manifestations in patients affected by cancer (Ronson, 2005). Through a review of psycho-oncological scientific literature, we examined some studies whose results demonstrate the existence in cancer patients of a relationship between trauma, dissociation, and emotional reactions to stress (e.g., Gershuny and Thayer, 1999; Maciejewski and Prigerson, 2013). Other authors show much more critical positions, highlighting the retrospective nature of the phenomena of dissociation, such as memory distortion, implemented even before the oncological pathology (Merckelbach and Muris, 2001).

With respect to the role of memory in the genesis of dissociative processes, Dunn et al. (1993) evaluated the information obtained from the patients after their oncologist had given them their cancer diagnosis, checking to what extent this was stored in their memory over the next 19 days. The results, showing that only 25% of the information given by the medical oncologist was remembered, allow the hypothesis of a dissociative response to receiving a diagnosis of cancer. In actual fact, the mnemonic gaps of the subjects could be attributed to dissociative amnesia, or to some other deficit related to different memory systems. However, this interpretation must be accompanied by caution as the research methodology does not allow the quality of the communication between doctor and patient to be known, the possible difficulties of understanding of some subjects or the activation of the mechanism of denial. With regard to the methodological limitations of various researches, and in particular of the above-described one, it is evident that these results undermine the traditional concept of dissociation, according to which this has an adaptive function, since the impact of a traumatic experience on the individual could be reduced through compartmentalization. Today, on the contrary, some of the most experienced clinicians in the field are putting forward a new idea of dissociation, stating that it is due to sudden changes in the networks of autobiographical memory (Ronson, 2005). To explore this concept of dissociation and its genesis, we herein report some research which, thanks to functional magnetic resonance imaging, has shown that at the time of communication of the diagnosis the brain areas involved in emotional responses are activated, interfering with the adequate processing of the information in the memory (Greene et al., 2001). In this regard, a pioneering research by Weisman and Worden (1976) conducted on 120 cancer patients showed the presence of peritraumatic dissociative symptoms, such as manifestations of alienation, depersonalization, derealization, distortion in perception of the self, and of the surrounding environment. Other authors have investigated post-traumatic reactions in cancer patients highlighting that, of the items in the IES, the one with the highest mean score is precisely the one that describes the perception of the experience as unreal (Tjemsland et al., 1996). Inevitably, cancer experience involves a fracture of the sense of continuity of the self and of one’s own existence. Indeed, many patients experience feelings of disbelief, numbness, unreality, out-of-body experiences, and sometimes even a feeling of a sudden inability to recognize, reporting that they are under the impression that their own body, feelings, and personality have changed forever (Brennan, 2001).

In the light of the above, the aims of this study were to: (1) evaluate the presence of cancer-related traumatic dissociative symptomatology in a sample of cancer patients; (2) examine the correlation of cancer-related dissociation and sociodemographic and medical variables, anxiety, depression, and post-traumatic stress symptomatology; and (3) investigate the predictors of cancer-related dissociation.

## MATERIALS AND METHODS

### PARTICIPANTS

The sample consisted of 92 general cancer patients (mean age 58.94, SD = 10.13; range 35–81 years) corresponding to the 92 questionnaires returned out of a total of 190 delivered. They were recruited from two hospitals in northern Italy: 53.3% of the returned questionnaires came from the centro oncologico ed ematologico subalpino (COES), Subalpine Center for Oncology and Hematology in Turin (*N* = 49) and 46.7% from the Ospedale Maggiore della Carità di Novara (Charity General Hospital) in Novara (*N* = 43). The study was approved by the Institutional Review Board at each participating institution and participating patients completed the informed consent process.

The sociodemographic variables were detected along with those relating to the state of the oncological pathology (see **Table 1**). The sample consisted of 55.4% women and 44.6% men.

**Table 1:** Summary chart showing the demographic descriptions of the sample.

	*N*	%
**Gender**
Male	41	44.6
Female	51	55.4
**Family status**
Married	59	60.9
Not married	5	5.4
Divorced	17	18.5
No answer	14	15.2

As regards their family status, 71.8% of the subjects were married (*N* = 56), 18.5% separated or divorced (*N* = 17), and 5.4% single or unmarried (*N* = 5), while 15.2% did not specify their marital status (*N* = 14). The cancers diagnosed included: lung, breast, gastro-intestinal, liver, skin, lymph node, bone, colon-rectum, urogenital, ovarian. With regard to the variables related to neoplastic disease, the sample was distributed as follows (see **Table 2**): 21.7% of patients were at stage I of the disease, 10.9% at stage II, 15.2% at III, and 32.6% at IV; while 78.3% had had surgery, and 82.6% chemotherapy.

**Table 2:** Summary chart showing the clinical descriptions of the sample.

	*N*	%
**Stage of the disease**
I	20	21.7
II	10	10.9
III	14	15.2
IV	30	32.6
No answer	18	19.6
**Cancer diagnosis**
Lung	8	8.7
Breast	24	26.1
Gastro-intestinal	21	22.8
Liver	7	7.6
Skin	1	1.1
Lymph node	2	2.2
Bone	4	4.3
Colon-rectum	12	13
Urogenital	4	4.3
Ovarian	3	3.3
No answer	5	5.4
**Chemo-therapy**
Yes	76	82.6
No	14	15.2
No answer	2	2.2
**Surgery**
Yes	72	78.3
No	18	19.6
No answer	2	2.2

The time elapsed since diagnosis ranged from 1 to 108 months, with an average of 15.54 months and SD = 16.46. On the basis of the data obtained using the Karnofsky Performance Status Scale, the psycho-physical state of the subjects on the whole was good, since with a range from 60 to 100 in the sample, the mean score was equal to 88.80%, with SD = 12.3.

### MATERIALS

The Karnofsky Performance Status Scale (KPS; Karnofsky and Burchenal, 1949) is a scale of health assessment in patients with malignant tumors, which evaluates the quality of life of a cancer or terminal patient through parameters related to activity limitation, self-care and self-determination. It measures the patient’s physical condition, performance, and prognosis following a therapeutic intervention.

The results may vary by a percentage ranging from 0 to 100%, where 100% reflects a good state of health, and 0% represents death.

The Hospital Anxiety and Depression Scale (HADS; Zigmond and Snaith, 1983) is a self-assessment questionnaire developed to detect states of anxiety and depression in patients suffering from organic diseases. This tool consists of two 7-item scales, one for anxiety assessment (HADS-A) and the other for depression assessment (HADS-D). For each statement, the patient is asked to indicate which of four possible options best describes his/her emotional state by means of a score between 0 and 3, in which 0 represents absence of the symptom and 3 its maximum severity (Bjelland et al., 2002). The score in both scales is the result of the sum of the scores assigned by the individual to each item.

The Impact of the Event Scale–Revised (IES-R; Weiss and Marmar, 1997; Italian version Giannantonio, 2003) aims to detect the presence of symptoms resulting from exposure to the specific traumatic event in the week before its administration. It investigates the ways the PTSD typically responds, since its three subscales evaluate the level of Intrusion (IES-R 1), Avoidance (IES-R 2), and Hyperarousal (IES-R 3) perceived by patient. The IES-R consists of 22 items on a four-score scale where 0 stands for “not at all,” 1 for “a little bit,” 2 for “moderately,” 3 for “quite a bit,” and 4 for “extremely.” The score is obtained by combining the three subscales, whose respective ranges are Intrusion, 0–32; Avoidance, 0–32; and Hyperarousal, 0–24. The cut-off is set at 33, while a score between 24 and 29 is taken as a sign of a partial PTSD and, finally, a score equal to or greater than 37 indicates a high presence of post-traumatic symptomatology (Weiss and Marmar, 1997).

The Peritraumatic Dissociative Experiences Questionnaire (PDEQ; Marmar et al., 2004) was developed with the purpose of investigating the dissociative component in response to a traumatic event. It therefore includes the detection of symptoms such as derealization, depersonalization, amnesia, altered perception of time, confusion, and impaired consciousness. It consists of 10 items, each of which has five alternative answers, whose score ranges between 0 (“not at all true”) and 5 (“extremely true”). The total score is obtained by adding up the scores obtained for individual items and thus lies between 0 and 50.

## RESULTS

For the statistical analysis, the Software Statistical Package for Social Science (SPSS) version 21.0 was used. The Pearson test and Student’s *t*-test were used in order to evaluate the correlation and to compare means between PDEQ scores and sociodemographic and clinical variables. A stepwise multiple linear regression model was used to analyze the relationship between the dependent variable (PDEQ scores) whenever any changes occurred in the clinical and socio-demographic conditions of the variables related to the psychological state. Values of *p* ≤ 0.05 were considered statistically significant.

### ANXIOUS AND DEPRESSIVE SYMPTOMATOLOGY

The anxious and depressive symptomatology was assessed by the HADS.

The average of the scores obtained in our sample is close to the cut-off score but still below the threshold. With regard to anxious symptomatology, the subjects obtain a score between 0 and 17, with an average of 7.07 (SD = 4.20), while with regard to the presence of depressive symptomatology, the results show a score between 0 and 19, with an average of 6.97 (SD = 4.87). The overall score resulting from the sum of the scores related to the two scales lies in a range between 1 and 33, with a mean score of 14.04 (SD = 8.45). 54.3% of the subjects achieved HADS scores indicating the presence of anxiety symptoms (*N* = 50), compared to the depressive symptoms found in 60.9% of the subjects (*N* = 56). Finally, considering the total score, it can be noted that in 55.4% of cases (*N* = 51) there is a score above the cut-off.

### POST-TRAUMATIC SYMPTOMATOLOGY

29.3% of the subjects (*N* = 27) reported mild post-traumatic symptomatology, while 32.6% had symptoms of moderate severity (*N* = 30). The analysis of the three subscales investigating the extent of intrusion, avoidance, and hyperarousal reported the following results: IES-R 1 (intrusion) scores in a range between 0 and 28, with a mean of 11.3 (SD = 6.62); IES-R 2 (avoidance) scores in a range between 0 and 26, with a mean of 10.50 (SD = 6.09); and IES-R 3 (hyperarousal) scores in a range between 0 and 19, with a mean of 7.28 (SD = 5.25). Finally, the IES-R total score lies in a range between 1 and 73, with a mean value of 28.89 and SD = 16.35.

### DISSOCIATIVE SYMPTOMS

The presence of peritraumatic dissociative symptoms in our sample was investigated through the PDEQ questionnaire. In particular, scores above the cutoff were found in 31.5% of subjects (*N* = 29). The scores obtained ranged between 8 and 40, with a mean score of 18.58 (SD = 8.27).

### PREDICTION OF PERITRAUMATIC DISSOCIATION

**Table 3** presents the correlation coefficients calculated between all the potential independent variables and PDEQ scores in order to select the optimal set of potential predictors of peritraumatic dissociation. This method was adapted from the methodology developed by Blanchard et al. (1996), which was used in the first systematic attempt to predict post-traumatic stress symptomatology following trauma in motor vehicle accidents.

**Table 3:** Correlations between the sociodemographic and clinical variables, and the dissociative peritraumatic symptomatology.

Variable	Peritraumatic dissociative experiences questionnaire (PDEQ) score
Months after diagnosis^a^	-0.169
Stage of disease^b^	0.149
Age^a^	-0.144
KPS	-0.041
HADS-A	0.476**
HADS-D	0.364**
IES-R 1	0.698**
IES-R 2	0.619**
IES-R 3	0.681**
IES-R total score	0.729**

^a^*N* = 90; ^b^*N* = 74; **p < 0.01.

No linear correlation was found between the presence of dissociative symptoms and the socio-demographic and medical variables (months after diagnosis and KPS score). On the contrary, the use of the Student’s *t*-test to compare the means of two independent samples highlighted the presence of a higher mean score relating to the scale of dissociation in patients receiving chemotherapy (*N* = 76, mean = 19.55, SD = 8.47) compared with patients who were not (*N* = 14, mean = 14.43, SD = 5.51; *p* ≤ 0.05). Having undergone surgery in relation to an oncological pathology does not mean higher PDEQ scores will be detected (*p* ≥ 0.05).

The results indicated that dissociative symptomatology was positively correlated with HADS scores (HADS-Anxiety: *r* = 0.476, *p* < 0.001; HADS-Depression: *r* = 0.364, *p* < 0.001) and with IES-R scores (IES-R-Intrusion: *r* = 0.698, *p* < 0.001; IES-R-Avoidance: *r* = 0.619, *p* < 0.001; IES-R-Hypervigilance: *r* = 0.681, *p* <0.001).

On the basis of the trauma literature, it was also deemed necessary to control the effects of demographic variables and treatment complications following diagnosis of cancer.

**Table 3** presents a summary of the variables that were significantly correlated with PDEQ scores.

A simultaneous multiple regression was conducted using the five variables correlating most strongly with the PDEQ scores (HADS-A, HADS-D, IES-R 1, IES-R 2, and IES-R 3).

In **Table 4**, the tolerance values smaller than 0.50 and VIF greater than 2 indicate suspected collinearity for the predictors. However, the values were acceptable with reference to the literature (e.g., Rogerson, 2001; Pan and Jackson, 2008), although the results should be considered with caution.

**Table 4:** Collinearity statistics.

Model		Tolerance	VIF
1	IES-R 1	1.000	1.000
2	IES-R 1	0.298	3.360
	IES-R 3	0.298	3.360
3	IES-R 1	0.257	3.892
	IES-R 3	0.288	3.476
	IES-R 2	0.485	2.063

The Durbin-Watson statistic, its value (2.398) slightly outside the range 1.5-2.2 suggested as acceptable, would seem to indicate a slight autocorrelation between errors and therefore suggests caution in interpreting the results. Through the stepwise regression, the model was perfected in three subsequent steps, each time increasing the number of predictors (at first only IES-R 1, then R3 and finally IES-R 1-IES, IES-R 3, and IES-R 2). As can be seen in **Table 5**, there is a progressive improvement in the data adjustment to the model as the multiple regression progresses: the final value of the coefficient of determination *R*^2^ = 0.539, adjusted to 0.523 (the small difference between the two indicates the absence of redundancy between the predictors) means that this model accounts for more than 50% of the dependent variables.

**Table 5:** Variables identified by stepwise regression analysis as predicting PDEQ scores.

	Model 1			Model 2			Model 3		
Variable	*B*	SE *B*	*B*	*B*	SE *B*	*B*	*B*	SE *B*	*B*
IES-R 1	0.869	0.095	0.696**	0.525	0.170	0.421**	0.391	0.180	0.313*
IES-R 3				0.515	0.214	0.329*	0.436	0.214	0.278*
IES-R 2							0.286	0.142	0.212*
R^2^		0.485			0.517			0.539
*F* for change in R^2^		82.872**			5.788*			4.071*

*p < 0.05; **p < 0.01.

## DISCUSSION

The majority of the sample group, about 62% of the subjects (*N* = 57), reported at least a mild form of post-traumatic symptomatology and 31.5% (*N* = 29) reported peritraumatic dissociative symptoms. Consistent with the results of Kangas et al. (2002); Hodgkinson et al. (2007), our findings highlight the absence of linear correlation between the presence of post-traumatic symptomatology and the socio-demographic and medical condition. With respect to this last point, in the literature it has been hypothesized that PTSD symptomatology is related to more advanced stages of the disease and types or length of cancer treatments, but the research findings are still ambiguous (Andrykowski and Kangas, 2010). In line with the studies of Granieri et al. (2013) and Pérez et al. (2014), we see our findings as underlining the role of subjectivity, resilience factors and personal history of the patient’s life, rather than dwelling on the objectivity of the cancer event in itself.

The relationship found between the dissociation scores and the parameters related to psychological complications (Anxiety, Depression, Intrusion, Avoidance, and Hyperarousal) can be seen as an indicator of suffering in cancer patients, in both the management of feelings and the creation of new meanings generated around the disease condition. Indeed, if dissociation can protect an individual from overwhelming emotions (van der Hart et al., 2004), it may also impede long-term recovery by preventing emotional processing and integration into the explicit memory of potentially traumatic memories, generating distortion or narrative fractures which may contribute to maintaining the symptoms in a circular way (van der Kolk and Fisler, 1995; Brown et al., 2014).

The linear regression model showed that 53.9% of the variance of our 3-factor model (Intrusion, Avoidance, and Hyperarousal) seems to be related to the post-traumatic symptomatology.

As can be seen from our results, only the three traumatic subscales had significant positive regression weights, indicating that patients with higher scores on these scales were expected to have higher dissociation scores. The sociodemographic and clinical variables did not contribute to the multiple regression model. These results allow us to hypothesize a specific psychological reaction which may be ascribed to the traumatic spectrum within the context of cancer, emphasizing the close relationship between the origin of dissociative constituents which, according to the scientific literature, compose the traumatic experience.

It is important to note that our study, like most researches in the context of PTSD and cancer (Andrykowski and Kangas, 2010), has used a cross-sectional design. Tautologically, this kind of study does not allow a longitudinal perspective, precluding the detection of different threats across disease and treatment progressions, and limiting what we can conclude about the direction of causality between the genesis of PTSD symptomatology and dissociation. For this reason, in agreement with Breh and Seidler (2007), it would be advisable also in the field of psycho-oncology to address future research to a clearer conceptualization of peritraumatic dissociation, as it is still unclear whether the latter is a symptom, a predictor or something else, in the framework of psycho-traumatic stress syndrome.

A second limitation regards the generalizability of the results. Since the number of cancer patients was limited and the sample was composed of volunteers, the findings may not be valid for the whole population. Future studies are needed to detect the aspects that can influence the etiopathogenesis of dissociation, as well as PTSD symptomatology during the cancer journey, referring to a diverse cancer population, and to a different stage of the disease and treatments. Finally, part of the dissociative symptomatology reported by patients (e.g., insomnia, irritability, fatigue) may be related to anti-tumor therapy. This variable has not been taken into account in this study, but it is important for researchers and clinicians to be aware, as observed by Kangas et al. (2002), that there are also differential diagnostic issues arising from cancer or treatment which induce physical symptoms that overlap with apparently dysfunctional psychological responses.

Despite these limitations and given the lack of longitudinal studies evaluating dissociative processes and PTSD symptomatology in the cancer context, our work provides a valuable perspective of these phenomena, helping clinicians in the planning phase of prevention and intervention programs, which should give patients the opportunity to openly express psychological and traumatic aspects of their experience. Specific and accurate psychological support can lead patients to a better assimilation of distressing unintegrated memories into coherent narratives, and to develop efficient coping strategies helping the patients to become better at recognizing and meeting their emotional and meaning-related needs.

## Conflict of Interest Statement

The authors declare that the research was conducted in the absence of any commercial or financial relationships that could be construed as a potential conflict of interest.
